# To sleep, perchance to dream

**DOI:** 10.3325/cmj.2020.61.475

**Published:** 2020-10

**Authors:** Charles H. Calisher

A few nights ago, I had a dream; almost everyone dreams. I often remember my dreams for a short time after I awaken. There is a great deal of literature about the supposed meaning of dreams, much of it originating with psychoanalytic theory. Sigmund Freud, Carl Jung, Alfred Adler, and numerous other outstanding psychiatrists, psychologists, neurologists, and other specialists investigated and published their hypotheses, intending to determine and clarify whatever dreams may signify. It is likely that none of these hypotheses is correct; certainly none has been proven (https://en.wikipedia.org/wiki/Psychoanalytic_dream_interpretation). It is clear that dreams are based in brain activity. Whether they reflect essentially random brain activity or more organized brain function is unclear, as is the function of this universal phenomenon. Perhaps dreams even contain useful and wonderful ideas, creations not recognized during waking hours. Dreaming occurs during the rapid eye movement (REM) cycle of sleep. One of our dogs twitches, barks, whines, and otherwise engages in behavior consistent with our experience of dreaming. Although other mammals are known to exhibit REM sleep, I have no idea if they dream or what they might dream about and I have no idea whether they dream as we do; elephants, bats, cats (whatever cats might dream about, I am certain it isn’t pleasant), maybe even hamsters, although hamsters do not do enough during the day to make it likely they need to review it. Anyway, if hamsters dream, I really do not care.

So, the other night I dreamed that my mother (who died in 1974) was lecturing me about being certain to fill my truck with petrol before I left for work. First of all, it was filled with petrol and second, I am retired, so do not go anywhere to work. Most importantly, my mother never lectured me while she was alive, so I found all this very unusual. Whenever I confront something even distantly psychological (Why do I want a beer whenever we have none in the refrigerator?), I confer with my wife, my skilled resident psychologist. Usually, she says, “I do not know. What happened to the beer in the refrigerator?” This time she said, “What happened to the beer in the refrigerator?” I obviously am then on my own.

When cogitating about my recent dream, it occurred to me that perhaps I had mistaken some other woman for my mother, but who could that have been? I once (just once, many years ago) consulted with a chiropractor about severe back pain I had been having. He took a full body x-ray, slapped it in a viewer, and asked me whether I had had a poor relationship with “a woman.” Let’s see: at one time or another I had a wife, mother, sister, grandmother, many girlfriends, women at work, women who worked in supermarkets, women editors, neighborhood women, and, of course, women scientific colleagues. I asked where the toilet was, left his office, went home, and never saw him again. Last I heard, he was working at a car wash.

Still, dreams fascinate me. Mine are always quite funny and appear to be disorganized, jumping from one apparent topic to another, juxtaposing seemingly unrelated people, events, situations, conversations, and problems, but all somehow related in and of themselves. Some years ago, at one phase of our investigations of the rodent-borne hantavirus, Sin Nombre virus, in southern Colorado, we were doing both field work and laboratory studies. These included collecting insects, mostly ground dwelling insects, as well as seeds and other tree products. One night I dreamed that ants (family Formicidae, order Hymenoptera), which are well known to contain formic acid, were urinating on our rodent traps! When I awoke, I began thinking about the urinary pH of rodents eating insects (in colder months) as compared with their urinary pH when eating grass seeds, grasses, and berries (in warmer months). I considered that their urinary pH might differ between seasons. Given that hantaviruses are sensitive to exposure to low pH, it occurred to me that rodents might have higher urinary pH in warmer months and lower urinary pH in colder months and that such differences might result in virus inactivation in colder months, accounting for the lower rate of virus transmission detected during the winter. That is, the rodents might be producing virus in their kidneys but that the virus might be excreted, but in an inactivated form.

We began a series of studies, first establishing a colony of deer mice (*Peromyscus maniculatus*), the principal rodent host of Sin Nombre virus, then feeding them either laboratory mouse chow, kale (*Brassica oleracea*), or dried, commercially available insects (mealworms, the larval form of the mealworm beetle *Tenebrio molitor*, and crickets, *Acheta domestica*). In sum, we determined that the urinary pH of deer mice fed laboratory chow was elevated (about 7.2) and remarkably uniform, the urinary pH of deer mice fed kale was slightly lowered (about 6.9), and the urinary pH of deer mice fed desiccated insects was relatively low (about 5.6). Switching these diets from kale to insects or insects to kale caused a reversion of these effects. Our hypothesis was that hantaviruses, which infect kidneys, among other organs, and which are shed via urine and saliva of infected rodents, are infectious in warmer months when transmission rates are highest but are less infectious or even non-infectious when shed during colder months, when their urinary pH is lower due to dietary effects.

Because this had been a relatively small study, I applied for a grant to obtain funding so that we could test the hypothesis with sufficient statistical power. Of course, the funding request was unsuccessful. However, I thanked my brain for the excellent idea and wondered whether I could find a graduate student or two who might be interested in carrying on these studies, but then I retired and that was that. I did not retire for lack of funding, I retired so that I could watch more baseball, but now this damned coronavirus has shut that down, at least for the time being. I go to sleep each night hoping that my brain will create a good idea that I will be able to retrieve the next morning, but all I can think of when I awake is breakfast.

Consider all the brain power of the nearly 8 billion people of the world at work dreaming. If they would just go to sleep earlier in the evening, sleep later in the morning, and be able to re-orient their thoughts away from petty jealousies, long-term anger at people who never did anything to them or to anyone else, earning more money than anyone needs, quit worrying about what is going on in a neighbor’s bedroom, and quit condemning people who attend an even more ridiculous house of worship than their own, perhaps the aggregate cumulative brain power might move humanity a bit forward.

“Every great dream begins with a dreamer. Always remember, you have within you the strength, the patience, and the passion to reach for the stars to change the world.” – Harriet Tubman

Final note: I am a scientist. I believe in data. Writing columns like this is not in my nature. Therefore, I could not have written it and I thank my brain for having done it without my help. My heart, lungs, liver, kidneys, spleen and other body parts go about their usual boring but essential business but my brain keeps on working, 24 hours a day, 7 days a week, 12 months a year. It is amazing in its ability to parasitize me.

